# Unilateral lateral rectus muscle advancement surgery based on one-fourth of the angle of consecutive esotropia

**DOI:** 10.1186/s12886-017-0658-1

**Published:** 2017-12-29

**Authors:** Jung Yup Kim, Soo Jung Lee

**Affiliations:** Department of Ophthalmology, Haeundae Paik Hospital, Inje University College of Medicine, 875 Haeundae-Ro, Haeundae-Gu, Busan, 612-896 South Korea

**Keywords:** Consecutive esotropia, Intermittent exotropia, Surgical effect, Unilateral lateral rectus muscle advancement

## Abstract

**Background:**

To evaluate the efficacy of unilateral lateral rectus muscle advancement surgery based on one-fourth of the angle of consecutive esotropia within 25 prism diopters (PD) occurring after bilateral lateral rectus muscle recession for intermittent exotropia.

**Methods:**

Medical records of 11 patients who underwent unilateral lateral rectus muscle advancement for consecutive esotropia from 2011 to 2014 and who were observed for at least 6 months after surgery were retrospectively reviewed. The change in angle of deviation from before to after consecutive esotropia surgery, as well as the success rate and surgical effect, were evaluated.

**Results:**

Preoperative esodeviation was −19.6 ± 4.7 PD [median − 20.0 PD, interquartile range (IQR) 9.0] at distance and −16.5 ± 7.4 PD [median − 18.0 PD, IQR 17.0] at near. The mean surgical amount of unilateral lateral rectus muscle advancement surgeries, based on one-fourth of the angle of consecutive esotropia, was 4.8 ± 1.1 mm [median 5.0 mm, IQR 2.0]. Of the 11 patients, 10 (91%) recovered to orthotropia or exodeviation within 8 PD. The surgical effects of unilateral lateral rectus muscle advancement were 3.3 ± 0.7 PD/mm [median 3.6 PD/mm, IQR 1.0] after 1 day, 3.7 ± 0.6 PD/mm [median 3.8 PD/mm, IQR 1.0] after 1 week, and 3.8 ± 0.7 PD/mm [median 3.8 PD/mm, IQR 1.5] after 6 months.

**Conclusions:**

Unilateral lateral rectus muscle advancement surgery based on one-fourth of the angle of consecutive esotropia within 25 PD was successful in all 11 patients. The surgical effect was significantly greater in unilateral lateral rectus muscle advancement than in primary lateral rectus muscle recession. Reduction in the amount of surgery should be considered carefully in unilateral lateral rectus muscle advancement for consecutive esotropia.

**Electronic supplementary material:**

The online version of this article (10.1186/s12886-017-0658-1) contains supplementary material, which is available to authorized users.

## Background

The recommended surgical approach for patients with overcorrection following strabismus surgery is dependent on whether eye movement is limited. For example, if eye movement is not limited, surgery on unoperated muscles should be performed as if it were the initial operation; whereas, if there are any limitations in eye movement, surgery should be performed on previously operated muscles (Cooper’s Dictum) [1]. For example, patients with consecutive esotropia, without any limitation of eye movement, and who have undergone bilateral lateral rectus muscle recession, usually undergo medial rectus muscle recession. Conversely, patients who have undergone unilateral lateral rectus muscle recession and medial rectus muscle resection usually undergo surgery on the fellow eye [2, 3].

Reoperation on unoperated new muscle has several advantages. Surgery is relatively easy to perform and predictable because the anatomical structure is normal without adhesion of surrounding tissues. However, it also has the disadvantage that the number of muscles available for surgery is reduced if another operation is needed in the future. Therefore, even if eye movement is not limited, reoperation on the previously operated muscles may be the preferred option. Reoperation on the previously operated muscles has the following advantages: the condition of the operated muscle can be confirmed; the patient and his/her guardians can avoid the psychological concerns associated with operation on a new muscle; and it leaves muscles available for additional surgery, although there is a risk of inaccuracy [4]. Thus, most current surgeons believe in exploring the operated muscle and assess its status before reoperation.

Factors to consider in determining surgical methods and extent in patients undergoing surgery for consecutive esotropia include uncertainty about the outcomes of the reoperation, each individual’s innervational and anatomical characteristics affecting alignment, the type of primary exotropia, accompanying disorders of eye movement, and angle of deviation. To date, however, there are no standardized guidelines for surgical method and extent. This study evaluated the effects of unilateral lateral rectus muscle advancement based on one-fourth of the angle of consecutive esotropia within 25 prism diopters (PD) without abduction defect after bilateral lateral rectus muscle recession for intermittent exotropia.

## Methods

The medical records of 11 patients who underwent unilateral lateral rectus muscle advancement surgery from 2011 to 2014 to treat consecutive esotropia after bilateral lateral rectus muscle recession for primary basic type intermittent exotropia and who were followed up for a minimum 6 months, were reviewed retrospectively. Records were excluded from analyses if the patient had a history of prematurity, had other ocular diseases or had neurologic diseases like mental retardation and cerebral palsy. The study protocol was approved by the Institutional Review Board of Inje University Haeundae Paik Hospital.

Gender, age at exotropia surgery, angle of exodeviation, surgical extent of bilateral rectus muscle recession, angle of deviation and stereopsis before and after consecutive esotropia surgery, amount and success rate of unilateral lateral rectus muscle advancement, and surgical effect (PD/mm) were investigated. Patients who had consecutive esotropia ≥ 10 PD at distance or near for more than 6 months after intermittent exotropia surgery underwent unilateral lateral rectus advancement [4–6].

The angles of deviation before and after primary exotropia and consecutive esotropia surgery were measured using alternate prism cover tests at 33 cm near and 6 m distance with correction of refractive errors if necessary. The angle of deviation was considered positive for exodeviation, and negative for esodeviation. Stereopsis was measured by the Titmus test and stereopsis worse than 100 arc/s was defined as poor.

All operations were performed under general anesthesia by a single surgeon. Surgical dosage of bilateral lateral rectus muscle recession was based on tables derived from Parks [7]. During unilateral lateral rectus muscle advancement for consecutive esotropia, the lateral rectus muscle was confirmed as being attached to the planned position of the first operation, with no slippage or resistance on a forced duction test.

The results of previous studies have suggested that the surgical effect was greater for advancement surgery for consecutive esotropia than for conventional rectus muscle recession [2–4]. Thus, when the difference in angle of deviation between distance and near was ≤8 PD, the amount of lateral rectus muscle advancement was set at 1/4 of the larger deviation of distance and near (Table 1, patients 1 to 9, e.g. surgical amount [3.5 mm] of patient 1 = 1/4 of distance deviation [14 PD]). When the difference was greater than 8 PD, the patient underwent surgery based on 1/4 of the mean angle of the deviation of distance and near to avoid overcorrection at fixation distance with smaller esodeviation (Table 1, patients 10 to 11, e.g. surgical amount [3.87 rounded to 4 mm] for patient 10 = 1/4 of average of distance [25 PD] and near [6 PD] deviation) (Additional file 1).

**Table 1:** Angle of consecutive esodeviation and surgical effect of unilateral lateral rectus muscle advancement

No.	BLR rec.	Consecutive esotropia		ULR advancement		Stereopsis (Titmus test, arc/s)		Deviation after 6 months	Surgical effect (PD/mm)		
	Amount (mm)	Far (PD)	Near (PD)	Amount (mm)	Laterality	Before	After		1 day	1 week	6 months
1	5.5	−14	−6	3.5	Right	100	80	Ortho/Ortho	1.7	2.9	2.9
2	6	−20	−25	6	Left	100	100	Ortho/Ortho	3.8	3.4	3.8
3	6	−20	−20	5	Left	800	400	Ortho/Ortho	4.0	4.0	4.0
4	6	−25	−20	6	Left	80	80	+4 / +4	3.4	3.8	4.6
5	5.5	−16	−8	4	Right	80	80	Ortho/Ortho	3.0	3.0	3.0
6	7.5	−25	−25	6	Left	50	50	+4 / +4	2.7	4.2	4.8
7	8.5	−16	−14	4	Right	100	80	Ortho/Ortho	3.8	3.8	3.8
8	6	−25	−18	6	Right	100	100	Ortho/Ortho	3.6	3.6	3.6
9	7	−14	−14	3.5	Right	100	100	Ortho/Ortho	4.0	4.0	4.0
10	7.5	−25	−6	4	Left	100	80	+8 / +8	3.9	4.9	4.9
11	6.5	−16	−25	5	Right	50	50	Ortho/ +8	2.9	2.9	3.1
Mean ± SD	6.6 ± 0.9	−19.6 ± 4.7	−16.5 ± 7.4	4.8 ± 1.1					3.3 ± 0.7	3.7 ± 0.6	3.8 ± 0.7
Median(IQR)	6.0(1.5)	−20.0(9.0)	−18.0(17.0)	5.0(2.0)					3.6(1.0)	3.8(1.0)	3.8(1.5)

*SD* Standard deviation, *IQR* Interquartile range, *BLR rec* Bilateral lateral rectus muscle recession, *ULR* Unilateral lateral rectus muscle, *PD* Prism diopters

(−) indicates esodeviation, (+) indicates exodeviation

The angle of deviation and limitation of eye movement were measured 1 day, 1 week, and 6 months after unilateral lateral rectus muscle advancement. Surgery was defined as successful when exodeviation at distance was ≤8 PD. The postoperative amount of correction was calculated as the difference in angle of deviation measured after surgery with that measured immediately before surgery. The effect of surgery was calculated by dividing the amount of correction deviation (PD) by the extent of surgery (mm) and expressed as PD/mm.

Continuous variables were compared using the Mann-Whitney U-test, and linear regression analysis was performed to assess the relationship between the amount and effect of surgery. All statistical analyses were performed using SPSS for Windows 17.0 (SPSS Inc., Chicago, IL, USA), with a *p* value < 0.05 defined as statistically significant.

## Results

The mean age of the 11 patients, 10 girls and one boy, at the time of bilateral lateral rectus muscle recession was 8.7 ± 2.8 years (range, 4 to 13 years) [median 9.0 years, interquartile range(IQR) 3.0]. All 11 patients had basic type intermittent exotropia. The average preoperative angle of deviation was 25.8 ± 6.4 PD (range, 14 to 35 PD) [median 25 PD, IQR 10.0] at distance and 27.7 ± 8.5 PD (range, 14 to 40 PD) [median 30.0 PD, IQR 15.0] at near, and the mean amount of bilateral lateral rectus muscle recession was 6.6 ± 0.9 mm (range, 5.5 to 8.5 mm) [median 6.0 mm, IQR 1.5] (Table 1) (Additional file 1).

Mean esodeviation on the first day after bilateral lateral rectus muscle recession were −12.2 ± 6.1 PD (range, −20 to 0 PD) [median − 14.0 PD, IQR 6.0] at distance and −4.9 ± 5.0 PD (range, −14 to 0 PD) [median − 4.0 PD, IQR 6.0] at near. The mean angle of deviation before surgery for consecutive esotropia was −19.6 ± 4.7 PD (range, −25 to −14 PD) [median − 20.0 PD, IQR 9.0] at distance and −16.5 ± 7.4 PD (range, −6 to −25 PD) [median − 18.0, IQR 17.0] at near. No patient showed limitation of eye movement. Mean surgical amount of the unilateral lateral rectus muscle advancement for consecutive esotropia was 4.8 ± 1.1 mm (range, 3.5 to 6.0 mm) [median 5.0 mm, IQR 2.0], based on 1/4 of the larger deviation when the difference in angle of deviation at distance and near was ≤8 PD, and 1/4 of the mean angle of deviation at distance and near when the difference was >8 PD.

The mean age of the 11 patients at the time of unilateral lateral rectus muscle advancement was 9.3 ± 3.0 years (range 4 to 14 years) [median 9.0 years, IQR 4.0]. Patients were followed-up for a mean 22.2 ± 17.6 months (range, 6 to 54 months) [median 17.0 months, IQR 23.0]. The average postoperative deviation was −2.2 ± 3.4 PD (range, −8 to 0 PD) [median 0 PD, IQR 6.0] on the first postoperative day, −1.3 ± 4.4 PD (range, −12 to 6 PD) [median 0 PD, IQR 4.0] at 1 week, and 2.2 ± 3.3 PD (range, 0 to 8 PD) [median 0 PD, IQR 4.0] at 6 months. None of the 11 patients showed limitations in ocular movement. Ten (91%) recovered to orthotropia or exodeviation ≤8 PD, whereas one (No. 11) experienced a recurrence of 14 PD of esodeviation after 3 months. Following alternative patch treatment and prism glasses, this patient recovered to orthotropia 12 months after surgery.

**Fig. 1:**
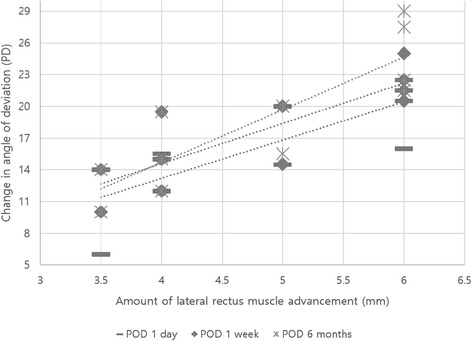
Linear regression analysis showing significant positive correlations between the amount of lateral rectus muscle advancement and change in angle of deviation at 1 day (R^2^ = 0.63, *P* = 0.004), 1 week (R^2^ = 0.70, *P* = 0.001) and 6 months (R^2^ = 0.74, *P* = 0.001) after surgery

The postoperative surgical effects of unilateral lateral rectus muscle advancement were 3.3 ± 0.7 PD/mm [median 3.6 PD/mm, IQR 1.0] at 1 day, 3.7 ± 0.6 PD/mm [median 3.8 PD/mm, IQR 1.0] at 1 week, and 3.8 ± 0.7 PD/mm [median 3.8 PD/mm, IQR 1.5] at 6 months (Table 1). Linear regression analysis showed that the extent of surgery was significantly and positively correlated with changes in deviation from before to after surgery, with *p* = 0.004 at 1 day, *p* = 0.001 at 1 week, and *p* = 0.001 at 6 months (Fig. 1).

We also analyzed a reference group of 115 patients who underwent bilateral lateral rectus muscle recession for treatment of intermittent exotropia during the same period, and had no recurrence or overcorrection for at least 6 months after surgery. The surgical effect in this group was 2.4 ± 0.4 PD/mm [median 2.4 PD/mm, IQR 0.5] on the first postoperative day, 2.0 ± 0.4 PD/mm [median 2.1 PD/mm, IQR 0.5] at 1 week and 2.0 ± 0.4 PD/mm [median 2.1 PD/mm, IQR 0.6] at 6 months. This surgical effect was significantly lower than that of unilateral rectus muscle advancement surgery at all three time points (*P* < 0.001 each). On the Titmus test, performed before unilateral lateral rectus muscle advancement for consecutive esotropia, 10 of the 11 patients showed preoperative stereopsis of ≤100 arc/s, whereas one had preoperative stereopsis of 800 arc/s, improving to 400 arc/s postoperatively.

## Discussion

A lack of standard guidelines has made determinations of surgical method and surgical amounts difficult for patients with consecutive esotropia.

Lee et al. [2] performed unilateral lateral rectus muscle advancement (average 6.7 mm) for consecutive esotropia within 25 PD (mean, 19.4 PD) in eyes with abduction limits, or in deviated eyes if abduction was not limited in either eye. In most of these patients, advancements were performed near the site of original insertion, whereas, in patients with esodeviation of 25 PD, advancements were 1.0 mm forward of the original muscle insertion site. These operations yielded good results, with a surgical effect of 2.7 PD/mm, which was greater than that of conventional lateral rectus muscle recession.

Kim and Son [3] reported that the mean surgical effect of bilateral lateral rectus muscle recession in 370 patients was 2.0 PD/mm. Seven of these patients experienced consecutive esotropia, with the mean surgical effect of lateral rectus muscle advancement in these seven patients being 4.3 PD/mm.

Shin et al. [4] performed bilateral lateral rectus muscle advancement to the original insertion site in patients having an angle of consecutive esotropia equal or greater than that of the first exotropia. In contrast, only one lateral rectus muscle was advanced to the original insertion site when the angle of consecutive esotropia was ≥5 PD less than that of the first exotropia. In that study, the surgical success rates of bilateral and unilateral lateral rectus muscle advancement were 56.3% and 100%, respectively, where surgery was considered successful when the angle of exodeviation was less than or equal to 8 PD after surgery. The surgical effects of unilateral lateral rectus muscle advancement were 2.9 PD/mm on the first postoperative day, 3.7 PD/mm at 6 months and 3.9 PD/mm (range, 3.3 to 4.7 PD / mm) at last follow up (≥24 months).

Kim et al. [8] reported that a mean surgical effect was 4.6 ± 0.9 PD/mm (range, 3.8 to 5.8 PD/mm) in a study of 11 patients who underwent unilateral lateral rectus muscle advancement.

Taken together, these studies show that the surgical effect of lateral rectus muscle advancement for consecutive esotropia is greater than that of conventional lateral rectus muscle recession. Therefore, in patients with consecutive esotropia, applying the same surgical amount for lateral rectus muscle advancement as would be used for lateral rectus recession will likely lead to increased rates of overcorrection.

In our study, the surgical effect of unilateral lateral rectus muscle advancement was very large in the patient with consecutive esotropia, similar to that of previous studies (Table 2). All patients showed esodeviation ≤8 PD on the first postoperative day, with mean esodeviations of −1.3 ± 4.4 PD at 1 week and 2.2 ± 3.3 PD at 6 months. Surgical effects after 1 day, 1 week, and 6 months were 3.3 ± 0.7 PD/mm, 3.7 ± 0.6 PD/mm and 3.8 ± 0.7 PD/mm, respectively, indicating a tendency of postoperative exodrift.

**Table 2:** Surgical effect of unilateral lateral rectus muscle advancement in other studies

No.	Authors	Reference number	Number of patients	Surgical effect (PD/mm)	Follow-up period after surgery
1	Lee JH et al., 2008	[2]	13	2.7	6 months
2	Kim JS and Son KH., 1995	[3]	7	4.3	8.6 months
3	Shin KH et al., 2014	[4]	9	3.9	24 months
4	Kim BH et al., 2014	[8]	11	4.6	1 week
5	Present study	[17]	11	3.8	6 months

*PD* Prism diopters

Assessment of a reference group of 115 patients who underwent bilateral lateral rectus muscle recession for intermittent exotropia showed mean surgical effects of 2.4 ± 0.4 PD/mm after 1 day and 2.0 ± 0.4 PD/mm after 1 week and 6 months. These effects differed significantly from those observed in the patients who underwent unilateral lateral rectus muscle advancement.

Because we previously observed a surgical effect of 2 PD/mm for lateral rectus recession, we selected a surgical extent of 1/2 the angle of exodeviation for patients undergoing unilateral lateral rectus recession. In applying the same surgical extent, 1/2 the angle of esodeviation for unilateral lateral rectus advancement, to patients undergoing surgery for consecutive esotropia, we observed frequent overcorrection. Based on studies showing that lateral rectus muscle advancement has a greater surgical effect in patients with consecutive esotropia, we set the extent of surgery at 1/4 the angle of consecutive esotropia [9–11].

In contrast to our findings, previous some studies [2, 8, 12] have reported that advancing the lateral rectus muscle to the original insertion site, which is slightly more forward than the expected extent of surgery, is safe. Uniform surgery to the site of original muscle insertion has also been advocated, regardless of the angle of consecutive esotropia, because the surgical effect is proportional to the angle of consecutive esotropia, and hence a greater angle of consecutive esotropia requires a greater reduction in surgical amount [13, 14].

In contrast, one study [4] reported that uniform advancement surgery to the original insertion site may be suboptimal, because the relationship between the angle of consecutive esotropia and the surgical effect is not strictly linear, with overcorrection being more frequent when the angle of primary exotropia is similar to the angle of consecutive esotropia and the latter has a moderate esodeviation of 20–30 PD. These findings suggested that the extent of bilateral lateral rectus advancement surgery should be reduced by 3–5 mm.

Because previously recessed muscles were found to gradually lose elasticity over time (Jampolsky’s leash effect) [15], moving these muscles back to their original position would restrain antagonistic muscles, resulting in exotropia. We calculated that the muscles in our 11 patients would require an average advancement of 6.6 ± 0.9 mm to return them to their original insertion position. However, surgery on our patients resulted in an average advancement of 4.8 ± 1.1 mm, or 1.8 ± 1.5 mm posterior to the original insertion site. This surgery yielded successful results without recurrence of exotropia.

However, three patients (No. 2, 4, and 8) underwent surgery to move the lateral rectus muscle to the original insertion site. These three patients had relatively small exotropia at the time of first exotropia surgery and a large angle of consecutive esotropia. These findings, as well as those of previous studies, suggested that the surgical effect on consecutive esotropia was greater than that of conventional exotropia surgery performed in accordance with Jampolsky’s leash effect [2–5, 15].

Other options for consecutive esotropia surgery could be considered, especially for large consecutive esotropia. Park SH et al. [16] compared unilateral lateral rectus advancement plus medial rectus recession with bilateral medial rectus recession for patients with 30–35 PD of large consecutive esotropia following bilateral lateral rectus muscle recession for intermittent exotropia. They suggested that both group showed successful surgical outcome after 12 months and advancement plus recession has advantage of preserving one medial rectus muscle for future intervention.

The present study had several limitations, including its retrospective design and the small number of patients (11 samples). Moreover, patients varied by gender, age and follow-up period. Future studies should be performed to evaluate the long term results of unilateral lateral rectus muscle advancement surgery in larger numbers of patients.

## Conclusions

Unilateral lateral rectus muscle advancement with the surgical dosage based on 1/4 of the angle of consecutive esotropia was successful, as assessed by both sensory and motor function, in patients who developed consecutive esotropia after bilateral lateral rectus recession. This surgery could be successfully performed even in eyes without limitations in eye movement. The surgical effect was greater than that of conventional rectus muscle recession. In planning lateral rectus advancement for correction of consecutive esotropia within 25 PD, it would be desirable to reduce the amount of secondary surgery compared with primary surgery. Unilateral lateral rectus muscle advancement based on 1/4 of the angle of consecutive esotropia could be useful for treatment of patients with consecutive esotropia.

## Additional file

Additional file 1: Supplementary file. (XLSX 13 kb)

## Abbreviations

BLR rec: Bilateral lateral rectus muscle recession
PD: Prism diopters
ULR: Unilateral rectus muscle
